# Fluorescence-based characterization of non-fluorescent transient states of tryptophan – prospects for protein conformation and interaction studies

**DOI:** 10.1038/srep35052

**Published:** 2016-10-17

**Authors:** Heike Hevekerl, Johan Tornmalm, Jerker Widengren

**Affiliations:** 1 Royal Institute of Technology (KTH), Dept Applied Physics, Experimental Biomolecular Physics, Albanova Univ Center, 106 91 Stockholm, Sweden

## Abstract

Tryptophan fluorescence is extensively used for label-free protein characterization. Here, we show that by analyzing how the average tryptophan fluorescence intensity varies with excitation modulation, kinetics of tryptophan dark transient states can be determined in a simple, robust and reliable manner. Thereby, highly environment-, protein conformation- and interaction-sensitive information can be recorded, inaccessible via traditional protein fluorescence readouts. For verification, tryptophan transient state kinetics were determined under different environmental conditions, and compared to literature data. Conformational changes in a spider silk protein were monitored via the triplet state kinetics of its tryptophan residues, reflecting their exposure to an air-saturated aqueous solution. Moreover, tryptophan fluorescence anti-bunching was discovered, reflecting local pH and buffer conditions, previously observed only by ultrasensitive measurements in highly fluorescent photo-acids. Taken together, the presented approach, broadly applicable under biologically relevant conditions, has the potential to become a standard biophysical approach for protein conformation, interaction and microenvironment studies.

Tryptophan (Trp) auto-fluorescence is widely used for label-free structural and dynamic studies of proteins^1^, and Room Temperature Phosphorescence (RTP) from the Trp triplet state can provide valuable complementary information^2^,^3^,^4^,^5^,^6^,^7^. In contrast to Trp fluorescence, which decays within nanoseconds after excitation, RTP lifetimes of Trp can vary from microseconds to seconds. This gives an extended time window for protein dynamics studies, and makes RTP a sensitive readout of local rigidity, environment and of exposure to intrinsic or extrinsic quenchers. However, compared to fluorescence, the phosphorescence signal is much weaker, and for useful RTP measurements all triplet state deactivation processes other than phosphorescence typically have to be suppressed. This requires careful sample deoxygenation and understanding of the mechanisms governing the Trp triplet state lifetime. The triplet state of Trp and other indole compounds have been extensively characterized by RTP^8^,^9^,^10^,^11^,^12^,^13^ and Flash Photolysis (FP)^14^,^15^,^16^,^17^, where the triplet state lifetime is determined by time-resolved triplet-triplet absorption measurements following light excitation. Depending on methodology, and most likely on differences in sample preparation, reported intersystem crossing rates of Trp, Trp triplet state lifetimes and quenching rates of different compounds have been found to differ by more than an order of magnitude^8^,^9^,^10^,^11^,^12^,^13^,^14^,^15^,^16^,^17^.

As an alternative to RTP and FP, Fluorescence Correlation Spectroscopy (FCS) can be used to determine population kinetics of triplet^18^ and other long-lived, non-fluorescent states of fluorescent molecules^19^,^20^,^21^, via changes in the detected fluorescence intensity from a limited number of fluorescent molecules at a time. For such studies, FCS combines the high environmental sensitivity and long observation time-window of the long-lived non-fluorescent states, with the high detection sensitivity following from the use of fluorescence as the readout signal. However, FCS measurements require the studied molecules to display high fluorescence brightness. Trp fluorescence is thus typically too weak for FCS studies, and has only been possible to use for crude diffusion studies of large proteins or aggregates, containing hundreds of Trps^22^,^23^.

In this study, we introduce transient state monitoring (TRAST) as a means to characterize non-fluorescent photo-induced states of Trp. In TRAST, the time-averaged fluorescence intensity from a sample subject to time-modulated excitation is recorded^24^. By analyzing how the recorded average fluorescence intensity varies with the excitation modulation characteristics, in particular the pulse duration, kinetic information of photo-induced, non-fluorescent transient states can be obtained. Like FCS, TRAST combines the detection sensitivity of fluorescence with the environmental sensitivity of long-lived non-fluorescent states. However, TRAST is not dependent on the detection of fluorescence fluctuations from individual molecules, and is therefore not restricted to studies of molecular species with high fluorescence brightness. In contrast to RTP, the fluorescence signal is relatively insensitive to impurities, and TRAST measurements do not require deoxygenation or careful control of quenchers. With relaxed requirements on sample preparation, detection quantum yield and time-resolution of the instrument, as well as on fluorescence brightness of the molecules studied, TRAST is broadly applicable, and has been demonstrated both for solution measurements^25^,^26^ and live cell studies^27^,^28^. This far, the studies have been based on fluorescence from added fluorophores. In this work, we introduce label-free TRAST spectroscopy in the ultra-violet wavelength range, applied to Trp transient state and protein conformation studies. By TRAST, we revisit the transient state kinetics of Trp in aqueous solutions, establish an electronic state model, investigate the influence of pH, oxygen, ascorbic acid, potassium iodide and various buffers, and compare the determined rates for triplet state formation/decay and photo-oxidation with those reported from RTP and FP measurements. Under acidic conditions, we identify a negative relaxation term in the detected fluorescence at onset of excitation, which we attribute to a delayed fluorescence anti-bunching, caused by excitation-induced proton transfer (EPT). Upon variation of pH and buffer concentration, the amplitude and relaxation time of this negative term was found to reflect the protonation state of Trp and the buffer-mediated recovery rates of Trp following EPT. Finally, TRAST was also demonstrated for protein studies. Conformational changes in a spider silk protein could be monitored via the triplet state kinetics of its Trp residue, reflecting its accessibility to molecular oxygen in a surrounding air-saturated aqueous solution. Taken together, our studies show that TRAST can record a whole set of transient state parameters, yielding environment-, protein conformation- and interaction-sensitive information, not retrievable via traditional protein fluorescence readouts. The presented approach offers a robust alternative to RTP, FP and traditional protein auto-fluorescence readouts for label-free micro-environmental monitoring, as well as for structural and dynamic studies of proteins, and is applicable under a broad range of biologically relevant conditions.

## Results

### Validation of Trp electronic state model –excitation irradiance and oxygen concentration dependence

**Figure 1:**
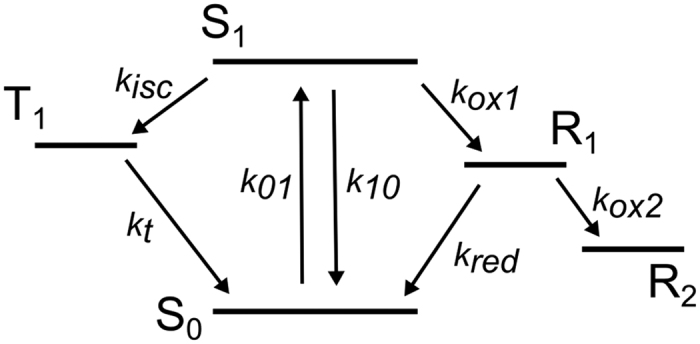
Basic electronic state model of Trp used in this study. S_0_ and S_1_ denote the ground and first excited singlet state, with the excitation and deactivation rates denoted by *k*_01_ and *k*_10_. From S_1_ intersystem occurs with a rate *k*_*isc*_ to the lowest triplet state, T_1_, which in turn decays with a rate *k*_*t*_ back to S_0_. Photo-ionization of Trp is assumed to take place from S_1_ with a rate *k*_*ox*1_. The formed radical, R_1_, can then either return to S_0_ via reduction (with rate *k*_*red*_), or go into a more long-lived non-fluorescent state R_2_. For Trp^14^,^15^,^16^,^17^, as well as for organic fluorophores in the visible range^21^,^36^,^38^, photoionization has been reported to be both mono- and bi-photonic in nature. In this study however, the applied laser excitation intensities were relatively low, and excitation to higher excited states, and subsequent bi-photonic ionization, can therefore be neglected. For fluorophores in general, subsequent photo-ionization to R_1_ may in principle also occur from T_1_, as reported e.g. for rhodamine dyes in polyvinlyalcohol^60^. In our experiments on Trp, this alternative photo-oxidation pathway is kinetically indistinguishable from S_1_ photo-oxidation (see Results). However, since previous studies of Trp photophysics have concluded that photo-oxidation takes place from S_1_ only^14^,^15^,^17^,^29^,^32^,^39^, we use this pathway of photo-oxidation in our model.

**Figure 2:**
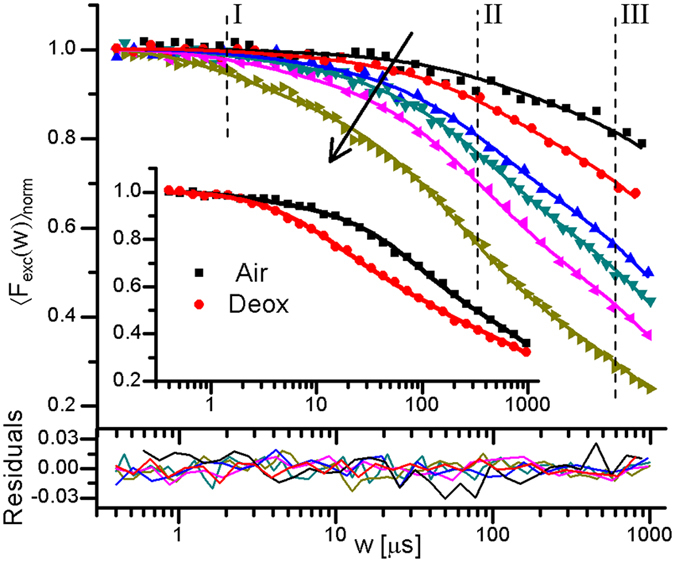
Normalized TRAST curves (dotted) recorded from a buffered 5 μM Trp solution (pH 7.4, 40 mM TRIS) using average *I*_*exc*_ of 4.8, 9.2, 14, 17, 28, and 65 kW/cm^2^ (black arrow indicate increasing *I*_*exc*_). Fitted curves (lines) generated as described in the main text, with residuals (bottom). Inset: TRAST curves recorded in air-saturated (black) and de-oxygenated (red) solutions, *I*_*exc*_ = 28 kW/cm^2^. Fitted curves (lines) generated as described in the main text.

Figure 1 shows the basic electronic state model used in this work. It is based on major features of Trp transient state transitions, as reported in literature^5^,^6^,^9^,^10^,^13^,^14^,^15^,^16^,^17^,^29^, and agrees with basic models used to analyse TRAST data of xanthene dyes in the visible wavelength range^25^,^26^. To validate the model, and to investigate how TRAST spectroscopy compares to RTP and FP for determining dark state transitions in Trp, TRAST curves of Trp in buffer solution were recorded under different excitation irradiances, under air-saturated and deoxygenated conditions (Fig. 2). In the TRAST curves (normalized time-averaged fluorescence intensity detected within an excitation pulse, 〈*F*_*exc*_(*w*)〉_*norm*_, plotted versus pulse duration, *w*, see Methods), three relaxation processes could be distinguished, within *w* = 2–3 μs (I), 60–100 μs (II), and in the sub-ms time range (III). These relaxation processes agree well with the three excitation-induced dark states in the electronic state model of Fig. 1, specifically since their amplitudes tend to increase and their relaxation times decrease with higher excitation intensities. Upon oxygen removal, a prominent increase of both the amplitude and the relaxation time of process (I) can be noticed in the TRAST curves (Fig. 2, inset), consistent with oxygen as an efficient quencher of the Trp triplet state^14^,^15^. Based on the rate equations and initial condition of Eq. 2, and using Eq. 9, rate parameters were numerically fitted to the TRAST curves (procedure described in the Methods section). The two curves (Fig. 2, inset) were fitted simultaneously, with the parameters *k*_*isc*_, *k*_*ox*1_, *k*_*red*_ and *k*_*ox*2_ set global for the curves, and only *k*_*t*_ allowed to be different between the curves. The fitted curves could well reproduce the experimental TRAST curves (inset Fig. 2, fitted parameter values in Supplementary Table 1). From this fitting procedure, *k*_*isc*_ was determined to 28 μs^−1^. A fluorescence lifetime of Trp, *τ*_*f*_ = 3 ns^30^,^31^, yields a triplet quantum yield of *q*_*T*_ = 0.084, which is in the middle of the relatively broad range of *q*_*T*_ values reported from RTP or FP studies: 0.0065^10^, 0.065^15^, 0.09^32^, 0.14^33^, 0.24^17^. *k*_*t*_ was determined to *k*_*t*_(*deoxy*) = 0.094 μs^−1^ in the deoxygenated solution, corresponding to a triplet decay time of *τ*_*T*_(*deoxy*) = 11 μs, and to *k*_*t*_(*air*) = 0.74 μs^−1^ in the air-saturated sample. Our value of *τ*_*T*_(*deoxy*), is in line with values obtained by FP (10 μs^15^, 14 μs^14^ and 43 μs^34^), while RTP lifetimes of Trp under similar conditions have been reported to be as long as 1.2 ms^8^. From the difference *k*_*t*_(*air*) − *k*_*t*_(*deoxy*) = 0.65 μs^−1^, and with approximately 0.24 mM of dissolved molecular oxygen in the air-saturated aqueous solution, we get a T_1_ quenching rate by oxygen of 2.7 ⋅ 10^9^ M^−1^s^−1^. This is somewhat lower than previously reported for Trp (~5 ⋅ 10^9^ M^−1^s^−1^ 14,15), but higher than for organic fluorophores (~2 ⋅ 10^9^ M^−1^s^−1^ 18). Given that the quenching rate for both the organic fluorophores and for Trp can be expected to be mainly determined by oxygen diffusion (D ~ 2 ⋅ 10^−5^ cm^2^/s 35), our determined *k*_*t*_ values seem reasonable. Oxygen, as an electron acceptor, can likely also promote the *k*_*ox*1_ rate, and a difference between the *k*_*ox*1_ rates in air-saturated and in deoxygenated aqueous solutions would then be expected. However, given a determined *k*_*ox*1_ rate in the range of 10^7^ s^−1^ (see below), the relative contribution to *k*_*ox*1_ from diffusion-controlled photo-oxidation of S_1_ by molecular oxygen is limited to a few percent and is within the experimental uncertainty of our measurements.

The TRAST curves of Fig. 2 were analyzed similarly to those in the inset, with all six curves fitted simultaneously. Given the generally low amplitudes of the relaxation process attributed to T_1_ relaxation (I), *k*_*isc*_ was fixed to 28 μs^−1^, as determined from the curves in the inset. *k*_*t*_, *k*_*red*_ and *k*_*ox*2_ were set global, and *k*_*ox*1_ was allowed to vary between the curves. The fitted curves could then well reproduce all the experimental TRAST curves, further confirming that the model in Fig. 1 adequately describes the observed dark state transitions of Trp. From the fitting, *k*_*t*_ = 0.71 μs^−1^, well in agreement with the value determined for the inset curves. *k*_*ox*1_ was determined within a relatively confined range for the different curves, from 5.7 to 9.4 μs^−1^, corresponding to a photo-ionization quantum yield of *q*_*ox*_ = 0.017–0.028. *k*_*red*_ = 0.011 μs^−1^ and *k*_*ox*2_ = 0.002 μs^−1^. Our determined *q*_*ox*_ values for Trp are considerably higher than for organic fluorophores in aqueous solution under comparable excitation conditions^36^. This may be a consequence of the higher excitation energies involved in the photo-induced electron transfer^37^. On the other hand, the determined *q*_*ox*_ range is lower than previously determined *q*_*ox*_ values of Trp by FP under comparable conditions (0.04^15^, 0.075^17^, 0.08^14^, 0.14^32^, 0.16^16^, 0.25^29^). This can partly be attributed to diffusion effects, with Trp in long-lived transient states in the detection volume being replaced by fresh Trp outside of the volume. Previous simulations of TRAST experiments^38^ have shown that state transitions slower than the probe molecule diffusion through the detection volume can lead to a two-fold under-estimation of the transition rates to a dark state (*k*_*ox*1_), and over-estimations of up to a factor of five of dark-state recovery rates (*k*_*red*_). Taking diffusion into account, we can thus estimate *q*_*ox*_ = 0.03–0.06, i.e. at the lower end among the previously reported values, and *k*_*red*_ to be as low as 2 ms^−1^ in the absence of Trp reductants in the solution. For comparison, we also fitted the TRAST curves in Fig. 2 and in the inset of Fig. 2 to a model, assuming photo-oxidation to occur also from T_1_, with the same rate as from S_1_. The quality of fit was as good as when assuming photo-oxidation from S_1_ only. This alternative model is kinetically indistinguishable from the model of Fig. 1, and results in considerably lower k_ox1_ rates (0.18 μs^−1^ and 0.025 μs^−1^, in the air-saturated and in the deoxygenated samples, respectively). However, since many studies of Trp photophysics favor photo-oxidation from S_1_ only^14^,^15^,^17^,^29^,^32^,^39^, we hereinafter used the model of Fig. 1 to analyse our data.

### Effects of reductants and quenchers

#### Ascorbic acid

**Figure 3:**
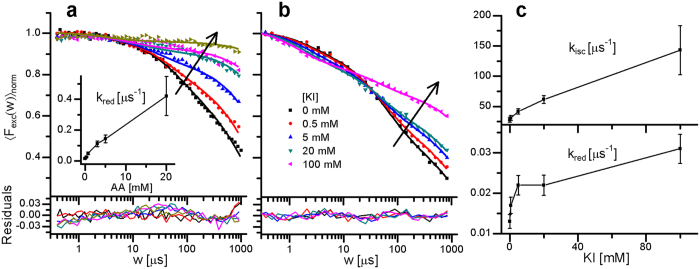
Normalized TRAST curves recorded from Trp in 40 mM Tris buffer, pH 7.4 (dotted). Fitted curves (lines) with residuals (bottom) generated as described in the main text. **(a)** Ascorbic acid (AA) titration, with [AA] from 0 to 20 mM. Black arrow indicates increasing [AA]. *I*_*exc*_ = 17 kW/cm^2^. Inset: Fitted *k*_*red*_ values vs [AA]. Error bars denote 95% confidence intervals. (**b)** Potassium iodide (KI) titration, with [KI] from 0 to 100 mM (*I*_*exc*_ = 65 kW/cm^2^). (**c)** Top: Fitted *k*_*isc*_ values vs [KI], Bottom: Fitted *k*_*red*_ values vs [KI], with error bars denoting 95% confidence intervals.

To verify that relaxation process (II) in the TRAST curves is mainly a consequence of Trp photo-oxidation, and to investigate how the photodynamics of Trp are affected by the presence of reducing agents, we added ascorbic acid (AA) in different concentrations ([AA] = 0–20 mM) and performed TRAST measurements at low excitation irradiances (*I*_*exc*_ = 17 kW/cm^2^) to suppress triplet state build-up. With higher [AA], a prominent reduction of the dark state build-up was evident from the recorded TRAST curves (Fig. 3a). Simultaneous fitting, with *k*_*isc*_ and *k*_*t*_ fixed to values determined above (28 and 0.74 μs^−1^, respectively), *k*_*ox*1_ and *k*_*ox*2_ set global for all curves, and with *k*_*red*_ allowed to take individual values for each curve, resulted in fitted curves well reproducing the experimental curves (fitted parameter values given in Supplementary Table 1). The globally fitted *k*_*ox*1_ and *k*_*ox*2_ values (10 and 0.0039 μs^−1^) agree well with the values determined for the curves in Fig. 2. The *k*_*red*_ parameter values, individually fitted to each TRAST curve, showed a close to linear dependence on [AA] (inset, Fig. 3a), with a quenching constant of *k*_*Qred*_(AA) = 2.5 ⋅ 10^7^ M^−1^s^−1^. This confirms that photo-oxidation strongly contributes to the dark state build-up of Trp in our measurements, that the photo-oxidized state R_1_ is sensitive to reducing agents, and indicates that relative changes of *k*_*ox*1_ and *k*_*red*_ can be accurately monitored. It can be noted that the *k*_*Qred*_(AA) determined for Trp is about two orders of magnitude lower than that determined for the organic dye Rhodamine 6G (Rh6G)^36^, and also that there is no obvious enhancement of photo-induced reduction upon addition of reductants in mM concentrations, as found for e.g. Rh6G^36^,^40^.

#### Potassium iodide (KI)

For organic fluorophores in the visible wavelength range, KI can influence both the triplet state and act as a fluorophore reducing agent, contributing to the recovery of photo-oxidized dyes back into viable fluorophores^40^. By the heavy atom effect, KI enhances *k*_*isc*_ for almost all dyes with excitation in the visible wavelength range, but can also enhance *k*_*t*_ by means of electron transfer for dyes with excitation maximum in the blue-green range^40^. To investigate how the transient states of Trp are affected, TRAST curves were recorded from Trp in buffer solution, with different KI concentrations added ([KI] = 0–100 mM) (Fig. 3b). KI was found to influence both the T_1_ and R_1_ kinetics of Trp. Simultaneous fitting, with *k*_*isc*_ and *k*_*red*_ allowed to take individual values for each curve, *k*_*ox*1_ and *k*_*ox*2_ set global for all curves, and with *k*_*t*_ fixed to the determined value (0.74 μs^−1^) for 0 mM KI and then assumed to increase linearly with [KI] with a quenching constant *k*_*Qt*_, resulted in fitted curves well reproducing the experimental curves (fitted parameter values given in Supplementary Table 1). In the fitting, a total quenching rate of S_1_ (*k*_*isc*_ included) by KI of 3.9 ⋅ 10^9^ M^−1^s^−1^, as reported from steady-state and time-resolved fluorescence measurements of Trp^10^, was included in the *k*_10_ rate. The individually fitted *k*_*isc*_ values show a close to linear dependence on [KI] (Fig. 3c), and linear regression yields *k*_*isc*_ + *k*_*Qisc*_·[KI] = 34 μs^−1^ + 1.1 ⋅ 10^9^ M^−1^s^−1^·[KI]. For *k*_*red*_ a less pronounced linearity was found. *k*_*Qt*_ was determined to 2.9 ⋅ 10^6^ M^−1^s^−1^. In comparison to bimolecular quenching constants determined for organic dyes in the visible range, the determined *k*_*Qisc*_ is similar as for Rhodamine Green (RhGr)^40^, but one order of magnitude lower than for Rh6G^18^. Similar to AA, *k*_*Qred*_ for KI is about two orders of magnitude lower than the corresponding quenching constant for a Rhodamine fluorophore (RhGr)^40^, and there are no indications of a photo-induced reduction of Trp upon addition of mM concentrations of KI, as observed for organic dyes in the visible range^40^. This observation, and the different *k*_*Qisc*_ and *k*_*Qt*_ between visible-range fluorophores themselves, and between these fluorophores and Trp, reflect differences in redox potentials and in S_1_ and T_1_ energy levels. Given the reported total quenching rate of S_1_^10^, our value of *k*_*Qisc*_ indicates that only a minor part of the KI-mediated quenching of S_1_ takes place via intersystem crossing to T_1_.

### Effects of pH and buffer concentration

#### pH effects in buffer solution

**Figure 4:**
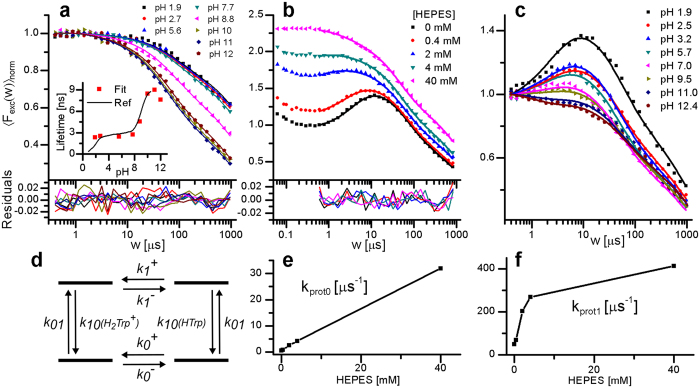
(**a**) TRAST curves recorded from Trp in a 40 mM TRIS buffer at different pH (dotted). *I*_*exc*_ = 14 kW/cm^2^. Fitted curves (lines) with residuals (bottom) generated as described in the main text. Fitted parameter values: *k*_*isc*_ = 15 μs^−1^, *k*_*ox*1_ = 8.6 μs^−1^*, k*_*red*_ = 13 ms^−1^ and *k*_*ox*2_ = 2.1 ms^−1^ (see text for details). Inset: The individually fitted values for 1/*k*_10_ (red circles), plotted vs pH together with the fluorescence lifetimes determined in refs 30 and 31 (black line). (**b**) Normalized average fluorescence intensity 〈*F*_*exc*_(*w*)〉_*norm*_ of Trp in H_2_O with different concentrations of HEPES (relative amplitudes between the curves preserved), pH 1.9, *I*_*exc*_ = 89 kW/cm^2^. Fitted curves (lines) with residuals (bottom) generated as described in the main text. (**c**) 〈*F*_*exc*_(*w*)〉_*norm*_ of Trp in H_2_O at different pH (1.9–12.4) in the absence of buffer, *I*_*exc*_ = 43 kW/cm^2^. (**d**) Protonation model for S_0_ and S_1_, added to the model of Fig. 1 in the analysis of the TRAST curves in Fig. 4b. (**e**) Protonation relaxation rate in S_0_, *k*_*prot*0_, determined from the TRAST curves in Fig. 4b, plotted versus the HEPES buffer concentration. Error bars denote 95% confidence intervals. (**f**) Corresponding protonation relaxation rate in S_1_, *k*_*prot*1_, plotted versus [HEPES]. Error bars denote 95% confidence intervals.

The influence of pH on the transient state population dynamics of Trp was investigated by recording TRAST curves from Trp in a 40 mM TRIS buffer over a wide pH range (1.9–12.1). The excitation irradiance was kept low (*I*_*exc*_ = 14 kW/cm^2^) to minimize triplet state build-up. In the recorded TRAST curves (Fig. 4a), the amplitude of the relaxation process II was found to be almost constant for pH 1.9–7.7, increase for pH 7.7–11.1 and then decrease slightly for pH 12.1. Rate parameters were fitted to the curves as above, based on the model of Fig. 1. The eight curves were fitted globally, but given the strong pH dependence of the *k*_10_ rate reported^30^,^31^,^32^, also the k_10_ rate was fitted, and was allowed to take individual values for each curve. The *k*_*isc*_, *k*_*ox*1_, *k*_*ox*2_ and *k*_*red*_ rates were globally fitted for all curves, and the *k*_*t*_ rate was fixed to 0.74 μs^−1^, as determined above. The fitted curves could well reproduce the experimental data (Fig. 4a), with the globally fitted rates (given in the figure caption) in reasonable agreement with those determined above, and with the individually fitted *k*_10_ rates found to comply very well with previously reported fluorescence lifetimes^30^,^31^,^32^ (inset, Fig. 4a). The variation of radical state formation with different pH can thus be explained by the strong pH dependence of the Trp fluorescence lifetime, *τ*_*F*_ = 1/*k*_10_, where higher *k*_10_ rates result in lower *q*_*T*_ and *q*_*ox*_ values.

#### pH- and buffer-dependent initial decay and fluorescence anti-bunching

For Trp in low pH (1.9) solutions, with no or sub-mM buffer concentrations present, an initial (<200–300 ns) decay in the TRAST curves is followed by a distinct negative relaxation term for *w* = 0.5–10 μs. Both the initial decay and the negative term were found to decrease in amplitude with increasing buffer concentrations (Fig. 4b) and pH (Fig. 4c). The pH dependence and time range of the initial decay suggest that it reflects formation of a previously reported, short-lived (~26 ns) triplet state of Trp^14^,^15^, protonated at its excited indole ring. The fast relaxation of this state was partly beyond the time resolution of our TRAST measurements, and we did not investigate the kinetics of this state further. Instead, we focused on the negative relaxation term, and analyzed the TRAST curves for pulse durations beyond this initial decay. Negative relaxation terms attributed to anti-bunching^41^ have previously been observed in single molecule fluorescence and FCS measurements^42^,^43^,^44^,^45^, with relaxation rates given by the sum of *k*_01_ and *k*_10_ (Methods, Eq. 4). For Trp, this would correspond to relaxation times in the ns time range, three orders of magnitude faster than what we observe. Given the clear dependence on both buffer concentration (Fig. 4b) and pH (Fig. 4c), the slow anti-bunching is likely due to excitation-induced proton transfer (EPT). EPT for Trp^39^,^46^,^47^,^48^ can take place due to different pK_a_ values of ground state Trp (S_0_), with pK_a_ values of 2.4–2.6^14^,^47^ and 9.4^14^,^49^, and excited state Trp (S_1_), with pK_a_* values not so precisely determined, but possibly shifted from the ground-state pK_a_ values by several pH units^1^,^49^,^50^. The slow anti-bunching was modelled by including reversible proton exchange for both S_0_ and S_1_, as depicted in Fig. 4d, into the model of Fig. 1. The *k*_10_ rates for H_2_Trp^+^ and HTrp were fixed to 1/(1 ns) and 1/(3 ns), respectively^30^,^31^. In the overall model, all other rates to and from S_0_ and S_1_ are assumed to be the same, irrespective of the state of protonation, in agreement with previous data for intersystem crossing^32^ and photo-oxidation^14^. The slow anti-bunching relaxation time at low pH and low buffer concentrations reflects the time it takes to reach a new equilibrium after onset of excitation, between the weakly fluorescent, double-protonated form H_2_Trp^+^ and the more fluorescent single-protonated form HTrp (Fig. 4b). The negative relaxation amplitude in the TRAST curves indicates that pK_a_ > pK_a_*, which at onset of excitation shifts the balance between HTrp and H_2_Trp^+^ in favor of HTrp, resulting in an increase in fluorescence intensity. The observed behavior of Trp, with a photon antibunching relaxation time far longer than seen in single-molecule experiments on fluorophores^41^,^42^,^43^,^44^,^45^ (Eq. 4), is similar to that recently observed in single-molecule experiments on highly fluorescent photo-acids^51^,^52^. Rate parameters were fitted to the curves in Fig. 4b, based on the model of Fig. 1, adding the possibility of proton exchange in S_0_ and S_1_ (Fig. 4d), with *k*_*t*_ and *k*_*isc*_ fixed to the values determined above (0.74 μs^−1^ and 28 μs^−1^), *k*_*ox*1_ and *k*_*ox*2_ fitted as global parameters, and *k*_*red*_ allowed to take individual values for each curve. Since the curves were recorded at the same pH (1.9) with different buffer concentrations, we assumed the ratios of the protonation on- and off- rates in S_0_ and S_1_, *k*_0_^+^*/k*_0_^−^ and *k*_1_^+^*/k*_1_^−^, to have common values. These ratios were thus fitted as global parameters for all curves. In contrast, the protonation relaxation rates for S_0_ and S_1_, *k*_*prot*0_ = *k*_0_^+^ + *k*_0_^−^ and *k*_*prot*1_ = *k*_1_^+^ + *k*_1_^−^, can be expected to vary with the buffer concentration and were fitted individually to each of the curves. The fitted *k*_*prot*0_ values show a linear dependence with *k*_*prot*0_ = 0.8·10^6^ s^−1^ + 0.8·10^9^ M^−1^s^−1^·[HEPES] (Fig. 4e). This indicates a diffusion-controlled proton exchange in S_0_ with a similar bimolecular rate constant as for e.g. fluorescein in buffer solutions^53^. In contrast, the buffer concentration dependence of k_prot1_ shows a more complex behavior. The fitted values for *k*_*prot*1_ are generally an order of magnitude higher than for *k*_*prot*0_ and tend to increase with higher buffer concentrations. The far higher, apparently not diffusion-limited, proton exchange rates within S_1_ may be explained by excitation-induced intramolecular proton transfer (EIPT), reported to take place from the protonated amino group of Trp to the excited indole ring^1^,^32^,^39^,^46^,^48^,^50^. However, transitions between rotamer states of Trp, having different potential surfaces in S_0_ and S_1_^54^ can also contribute to the observed behavior. The ratios *k*_0_^+^*/k*_0_^−^ and *k*_1_^+^*/k*_1_^−^ were globally fitted to 8.7 and 0.15, which with a pH of 1.9 yields an estimated pK_a_ of 2.8 and a pK_a_* of 1.1. The pK_a_ value is a bit higher than previously reported (2.6^47^, 2.4^14^), but the difference pK_a_-pK_a_* = 1.7 is in line with suggested differences between the S_0_ and S_1_ states of Trp^1^,^49^,^50^. The other rate parameters fitted for the curves in Fig. 4b were found to be well in agreement with the previously determined parameter values (Supplementary Table 1). For the highest buffer concentration studied (40 mM), the influence of the proton exchange in S_0_ and S_1_ on the TRAST curves is negligible, which supports that it can be neglected in the analysis of the TRAST curves in Fig. 4a (recorded at 40 mM TRIS buffer).

#### pH-dependence in absence of buffer

In the set of TRAST curves in Fig. 4c, measured at different pH with no buffer added, the negative anti-bunching relaxation observed in the TRAST curves at lower pH gradually disappears with higher pH, and for TRAST curves recorded at high pH a small positive relaxation process is instead observed in the μs time range. The observed general trend cannot be fit into our simple model, and quantitative curve fitting of the TRAST curves based on a more advanced model goes beyond the scope of this work. However, the observed trend can likely be understood as a combination of different pK_a_ values of S_0_ and S_1_, presence of EIPT and possible transitions between different Trp rotamer states. The molecular brightness, or *q*_*f*_, of Trp is pH-dependent and closely follows the variation of τ_f_, as depicted in Fig. 4a 30,31,47, with distinct increases in *q*_*f*_ at pH ~ 2.5 (pK_a_ of the COOH group of Trp) and at pH ~ 9.4 (pK_a_ of the NH_3_^+^ group of Trp). For the low pH transitions (H_2_Trp^+^ - HTrp), we concluded that pK_a_ > pK_a_^*^. Onset of excitation then favors HTrp over H_2_Trp^+^, and with *q*_*f*_(HTrp) > *q*_*f*_(H_2_Trp^+^) a relaxation term with negative amplitude in the TRAST curves can be generated. In the mid-pH range (2.5 ≪ pH ≪ 9.4), a negative relaxation term in the μs time-range is still observed in the TRAST curves (Fig. 4c). This cannot be attributed to differences in pK_a_ and pK_a_^*^, which are both far outside this pH-range. However, also in this pH-range, there is clear evidence for several Trp excited state reactions, including proton transfer, electron transfer and rotamer interconversions^55^. EIPT (from the NH_3_^+^ group to the excited indole ring of Trp) have been reported^32^,^46^,^47^ and may arise as a consequence of an increase of the pK_a_ of the indole nitrogen upon excitation. The efficiency of EIPT can depend on the rotamer state of Trp. Equilibria between different Trp rotamer states have been found to be different in S_0_ and in S_1_^54^,^56^, and if excitation favors rotamers with higher fluorescence quantum yields, this can result in the pH-dependent negative relaxation terms observed in our measurements. However, it should be noted that despite extensive studies, the transitions between different Trp rotamers and how they influence the fluorescence properties of Trp are still not fully understood^55^. A more precise explanation of our observations in the mid-pH range based on these transitions is thus difficult to give and is also beyond the scope of this study. For pH > 9.4, above the pK_a_ of the NH_3_^+^ group, EIPT from NH_2_ does not occur, and as a consequence no negative relaxation term is observed in this pH range (Fig. 4c). At this pH range however, transitions between HTrp and Trp^−^ can occur, and *q*_*f*_(Trp^−^) > *q*_*f*_(HTrp). The decreased fluorescence at onset of excitation, with a positive relaxation term in the μs time-range in the TRAST curves (Fig. 4c), may suggest that pK_a_ < pK_a_^*^ for this transition. This agrees with a previously estimated pK_a_^*^ of 12–13^1^, and that the less fluorescent HTrp is then favored over Trp^−^ at onset of excitation. However, triplet state build-up can also not be fully excluded as a reason for this positive relaxation term.

### Protein measurements

Given the sensitivity of the Trp transient states to the immediate environment, as displayed from the TRAST experiments, we investigated to what extent this could be exploited to monitor protein conformational changes.

TRAST experiments were performed on the N-terminal (NT) domain of the Major ampulate spidroin (MaSp), an extensively characterized spider silk protein^57^. NT contains ~130 residues and a single Trp residue buried in its core. MaSp can transform into insoluble fibres, induced by a pH change (pH 7 → 6) in a well defined region of the silk glands^56^. In this process, NT is proposed to act as a pH-regulated relay, conferring solubility at high pH and facilitating fibre formation at low pH. NT is mainly monomeric at pH 7 and dimeric at pH 6, and upon monomer to dimer interconversion the single Trp residue relocates from the inner core to a more solvent-exposed environment^57^.

**Figure 5:**
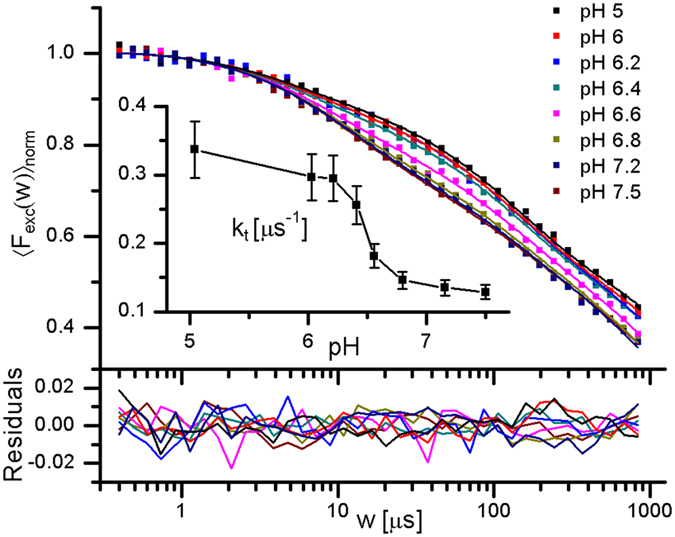
TRAST curves recorded at moderate excitation irradiance (*I*_*exc*_ = 9.2 kW/cm^2^) from a sample of 4 μM NT dissolved in 20 mM TRIS, with pH varied from 5 to 7.5. Fitted curves (lines) with residuals (bottom) generated as described in the main text. Inset: Fitted *k*_*t*_ rates, plotted versus pH. Error bars denote 95% confidence intervals.

We monitored this interconversion of NT by TRAST experiments, performed over a pH range of 5–7.5 (Fig. 5). For pH 7.5–7.2, the recorded TRAST curves were close to identical, displaying two major decay processes: a first process with a lifetime of 5–10 μs and a second, slower process present for *w* > 100 μs. With further reduced pH, from 7.1 to 6.1, lower amplitudes and faster decays for the first decay process were observed. For pH 6 and lower, again close to identical curves were recorded. The observed differences were found to be reversible, as the TRAST curve at pH 7.2 (black curve in Fig. 5), recorded after the low pH measurements, fully resembled the initial TRAST curves recorded in the upper pH range. All TRAST curves were subject to global analysis, based on the model of Fig. 1, with *k*_*isc*_, *k*_*ox*1_ and *k*_*red*_ fitted as global parameters for all curves, and *k*_*t*_ and *k*_*ox*2_ allowed to take individual values for the different curves. The fitted curves were found to well reproduce the experimental curves (Fig. 5). While the determined values of *k*_*ox*1_ (16 μs^−1^) and *k*_*red*_ (7.3 ms^−1^) were relatively similar to those for free Trp in aqueous solution (Fig. 2a), *k*_*isc*_ was found to be about a factor of four higher (99 μs^−1^). In proteins, local rigidity and quenching moieties close to the site of the indole ring have been found to strongly affect Trp phosphorescence lifetimes^5^,^11^,^58^. The influence of these factors on *k*_*isc*_ is difficult to investigate by RTP measurements, and also with FP, where the small changes in the transmitted optical radiation make it difficult to resolve different contributions and their kinetics^11^. However, these factors are believed to also influence the phosphorescence quantum yield, including *k*_*isc*_^6^. Together with the increased rigidity experienced by Trp when confined with peptide bonds in the protein, this can explain the higher *k*_*isc*_ observed for NT, compared to that found for free Trp in aqueous solution. The *k*_*t*_ rates, individually fitted to each of the TRAST curves, are plotted versus pH in the inset of Fig. 5. For low pH, with NT in a dimeric form and with Trp at the solvent-exposed surface, *k*_*t*_ ~ 0.4 μs^−1^. In the higher pH range on the other hand, with NT in a monomeric form and with Trp buried in the interior of the protein, *k*_*t*_ is significantly lower (0.15 μs^−1^). In the mid-pH range, *k*_*t*_ smoothly decreases with higher pH, with a half-range transition between the states at pH ~ 6.4. This is perfectly in agreement with a transition between the monomeric and dimeric form of NT at a pH of 6.1–6.5 (depending on the salt concentration), as determined by a combination of CD, NMR, Fluorescence cross-correlation spectroscopy and steady-state fluorescence measurements^57^. The found pH dependence of *k*_*t*_ is also well in agreement with the structural changes predicted for NT^57^, where the changes in *k*_*t*_ most likely can be attributed to changes in oxygen accessibility upon protein conformational changes. At the higher pH, the low *k*_*t*_ values indicate that oxygen quenching of the buried Trp is limited. At lower pH, *k*_*t*_ approaches 0.4 μs^−1^, in agreement with an expected higher oxygen quenching of the Trp triplet state at the protein surface. This *k*_*t*_ rate is approximately half of that for free Trp in air-saturated aqueous solution (Fig. 2a). This complies with an expected 2π solid angle of exposure of Trp to the solution, when at the protein surface, compared to a 4π solid angle when free in solution. Also for the values of *k*_*ox*2_, individually fitted to each curve, a transition in the rate values was found, with a mid-transition pH coinciding with that for *k*_*t*_. This likely also reflects the monomer to dimer transition of NT. However, a similar transition can also be seen for *k*_*red*_ and *k*_*ox*1_, if fitted individually instead of *k*_*ox*2_. One can therefore not directly assign this transition to a specific physical change in any of the *k*_*ox*1_, *k*_*ox*2_ or *k*_*red*_ rates. As a verification that the observed changes were caused by a protein conformation change of NT, and not by any pH influence on the Trp residue itself, only a minor increase in 〈*F*_*exc*_(*w*)〉_*norm*_ was found for free Trp in 40 mM TRIS buffer over the same pH range (Fig. 4a), and mainly at *w* > 1 ms, i.e. not in the time range of the triplet state relaxation.

## Discussion

In this study, transient state transitions of Trp in aqueous solution were determined by TRAST measurements. Starting from a simplified electronic state model, sets of TRAST curves were globally analyzed, with most of the rate parameter values either fixed or set global, and allowing model flexibility and parameter inter-relationships to be included. With a robust experimental approach, and analyses of the recorded curves with a limited number of free variables, transition rates of triplet and photo-oxidized states were identified and determined under different environmental conditions. Taking analyte diffusion into account for the slower transitions, all determined rates were found to be within the range of parameter values reported from FP and RTP measurements. For slower transitions already known, the influence of diffusion on the related relaxations in the TRAST curves can also provide a measure of the mobility of the analyzed molecules^59^. TRAST measurements can thus retrieve similar, and in some aspects extended, transient state information compared to RTP and FP, and can be performed under a broad range of conditions. The instrumentation is relatively simple, and can be further simplified, e.g. by replacing the frequency-tripled UV laser used for excitation in this work with a turn-key CW laser. Apart from the kinetics of triplet and photo-oxidized states, our TRAST measurements also revealed a pH- and buffer-dependent anti-bunching in the Trp fluorescence, previously only observed in specific, highly fluorescent photo-acids, requiring single-molecule or FCS measurements^51^,^52^. With its relatively much weaker fluorescence, anti-bunching in Trp fluorescence, or any transient state kinetics of Trp, is practically impossible to observe by FCS and single-molecule measurements. By TRAST, the observed anti-bunching can likely also be found in weakly fluorescent compounds other than Trp, having different pK_a_ and pK_a_^*^ values, providing information about local pH and buffer conditions.

In the NT protein measurements, the triplet state kinetics of its Trp residue was found to reflect the conformational state of the protein in a sensitive and accurate manner. Apart from offering a substitute to FP and RTP, this shows that Trp TRAST measurements can provide additional, highly environment sensitive parameters, extending the information available from traditional protein auto-fluorescence studies. Taken together, based on a robust and simple approach, applicable under a broad range of biologically relevant conditions, Trp TRAST measurements have the potential to become a standard biophysical approach for protein conformation, interaction and microenvironment studies.

## Methods

### Population dynamics in the electronic state model of Trp at onset of excitation

In the electronic state model of Fig. 1, only S_1_ generates fluorescence, upon decay to S_0_, and the emitted fluorescence intensity, *F*(*t*) is thus proportional to the S_1_ population, *S*_1_(*t*):


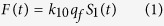


with *q*_*f*_ denoting the fluorescence quantum yield. For a Trp molecule subject to constant excitation starting at *t* = 0, and with the electronic state model in Fig. 1, the population probabilities of the electronic states can be described by:


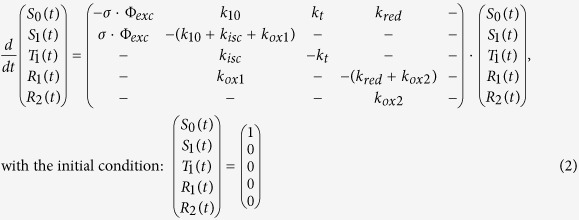


Here, *σ* denotes the excitation cross section, and Φ_*exc*_ the excitation photon flux, with 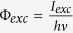, where *I*_*exc*_ is the excitation intensity and *hν* the excitation photon energy, so that *k*_01_ = *σ* ⋅ Φ_*exc*_. *S*_1_(*t*) can then in a general form be described by:


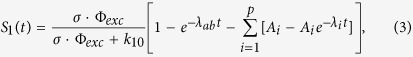


where *p* denotes the number of different photo-induced non-fluorescent states involved (states other than S_0_ and S_1_). *λ*_*ab*_ and *λ*_*i*_ are the eigenvalues, i.e. the rates of the relaxation modes of *S*_1_*(t)* upon onset of constant excitation, and *A*_*i*_ the related amplitudes, reflecting the population build-up of the different photo-induced non-fluorescent states. For most fluorescent molecules, equilibration between *S*_0_ and *S*_1_ after onset of excitation takes place within the time range of the fluorescence lifetime (ns), while the dark state relaxations (1/*λ*_*i*_) typically occur on a μs-ms time scale. The *S*_0_-*S*_1_ equilibration time, referred to as the anti-bunching time^41^,^42^,^43^,^44^ τ_ab_ = 1/*λ*_*ab*_, is typically given by^45^:


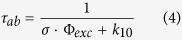


Based on Eq. 3, *λ*_*i*_ and *A*_*i*_ can be described analytically, as functions of the rate parameters of the model in Fig. 1 20,27,28,40.

### Transient State (TRAST) spectroscopy

In TRAST spectroscopy, the population dynamics of non-fluorescent, long-lived transient states of fluorescent molecules in a sample is determined from the average fluorescence intensity from the sample, when subject to different excitation pulse trains. We applied square-wave excitation pulse trains in a confocal setup, as described below. For a sample of fluorescent molecules, subject to an excitation pulse train with *n* pulses of duration *w* and period *T*, the detected time-averaged fluorescence, 〈*F*(*w*)〉, can then be expressed as^24^,^25^,^26^,^27^,^28^:


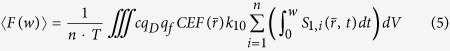


Here *n·T* is the total duration of the excitation pulse train, *c* the concentration of the fluorescent species, *q*_*D*_ the overall detection quantum yield of the instrument, and *CEF* the collection efficiency function of the confocal setup. The term 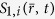 denotes the probability that a fluorescent molecule, located at 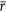 in the confocal detection volume, is in its excited singlet state at time *t* after on-set of the *i*:th excitation pulse. Dividing 〈*F*(*w*)〉 with the pulse train duty cycle (*η* = *w*/*T*) yields the average fluorescence intensity within an excitation pulse:


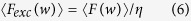


〈*F*_*exc*_(*w*)〉 normalized to 1 for pulse durations |*λ*_*ab*_| ≪ *w* ≪ |*λ*_*i*_|, denoted 〈*F*_*exc*_(*w*)〉_*norm*_, represents the averaged population of S_0_ and S_1_, within the pulse duration, and over the detection volume:


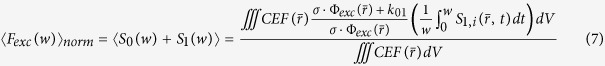


With knowledge of 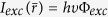, *CEF*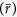, and the electronic state model (Eq. 2), and by use of Eq. 7, the pulse duration dependence of 〈*F*_*exc*_(*w*)〉, making up a so-called TRAST-curve, can be used to extract the transient state rate parameters, as previously shown for organic fluorophores^24^,^25^,^26^,^27^,^28^. Approximating the average excitation rate in the detection volume by:


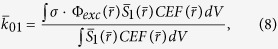


where 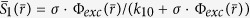, i.e. the S_1_ population at onset of excitation, after equilibration between the singlet states, but before dark state build-up. A simplified expression for 〈*F*_*exc*_(*w*)〉 can then be obtained, given by


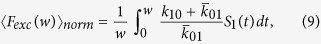


with *S*_1_(*t*) given by Eqs 2 and 3, or modified according to any other electronic state model than in Fig. 1.

### Instrumentation and measurement procedure

TRAST measurements were performed on a home-built, epi-illuminated, confocal microscope setup, with excitation from a mode-locked Ti:Sapphire laser (Mira 900, Coherent, Inc., pumped by a Nd:Vanadate laser (Verdi^TM^ V-10, Coherent, Inc.), operating at 870 nm, pulse width ~120 fs FWHM, 76 MHz repetition rate), frequency-tripled with a third harmonic generator (INRAD M/N 5-050, Inrad Inc.) to 290 nm. The mirrors (BHR-2506U-280, Lambda Research Optics Inc.) between the third harmonic generation system and the microscope also served as an excitation filter (reflection at 245–330 nm). The frequency-tripled laser beam was adjusted with a continuously-variable neutral density filter (Thorlabs), intensity-modulated by an acousto-optic modulator (AOM; MQ200-B80A1-266, AA Opto-Electronics, Orsay, Cedex), and was then focused into the sample by a microscope objective (Ultrafluar 100/1.25 glyc, Zeiss). From FCS measurements of 2-aminopurine in aqueous solution, the 1/e^2^ radius of the laser beam in the focal plane was determined to 445 nm. Fluorescence from the sample was collected through the same objective, separated from excitation light by a dichroic beamsplitter (FF310-Di01, Semrock, Inc.), focused onto a 75 μm diameter pinhole in the image plane and was then spectrally filtered (357/44 nm bandpass filter, Semrock). The fluorescence was then split by a polka dot beamsplitter (50:50, Thorlabs), focused and then detected by two single photon counting photomultiplier tubes (H7360-02, Hamamatsu), the signals of which were recorded with a PCI-6602 counter/timer card (National Instruments Corp.) for subsequent TRAST analysis (see below).

Square-wave excitation pulse trains were applied in the setup described above. TRAST curves were generated by recording 〈*F*_*exc*_(*w*)〉 for up to 30 different excitation pulse trains, with the pulse width *w* varied between 60 ns and 10 ms. The height of the pulses, that is the excitation irradiance, was kept at a constant level, as indicated below. The duty cycle *η* was kept at 1% to ensure a complete relaxation of tryptophan to the ground singlet state and/or renewal of tryptophan by diffusion at the onset of the next excitation pulse. The total duration of each excitation pulse train *n* ⋅ *T* was between 1 and 10 s.

### Data analysis

Data analysis was performed using software implemented in Matlab. The experimental data was pre-processed by subtracting detector dark counts and adjusting for detector dead time. Drifts in sample concentration, arising from a combination of bleaching and evaporation were corrected for by repetitive reference measurements throughout each experiment, using the shortest available pulse width to avoid buildup of any dark states. For the experiments with tryptophan in solution, the effect of bleaching was negligible and the evaporation in most cases small, about 0.5%. For certain longer measurements however, the total evaporation was as large as 5%.

In order to fit the data to a chosen photophysical model, simulated TRAST curves were generated by calculating 〈*F*_*exc*_(*w*)〉 from Eq. 5 for each pulse width, *w,* then normalized to 〈*F*_*exc*_(*w*)〉_*norm*_ (Eqs 6 and 7), if not stated otherwise. The excitation rate, 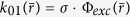, using an excitation cross section of *σ* = 1.26 ⋅ 10^−17^ cm^2^, can be estimated assuming a three-dimensional Gaussian distribution of 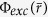, with a beam radius (1/e^2^) of 454 nm and an axial extension of 3 μm. However, the benefits of including a full 3D Gaussian excitation beam, with 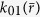 varying throughout the confocal volume, together with the microscope collection efficiency function *CEF*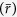 were found to be small. Great improvement in computational speed, without sacrificing much accuracy, could be achieved by instead approximating an average excitation rate in the detection volume, 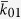, given by Eq. 8.

The remaining model parameters were then optimized using Eq. 9, and an iterative non-linear least square approach to match the calculated curves to the experimental data. Multiple TRAST curves could also be fitted simultaneously, with each rate being specified as global or independent between curves.

The simulations also used an adjusted value for the pulse widths, *w*. A small but constant shift in pulse duration, Δ*w*, caused by non-zero rise- and fall-times of the AOM, lead to a relative error in illumination time given by Δ*w*/*w*. With Δ*w* in the order of 10 ns, and constant for all pulse widths, this effect is only relevant for the shortest pulses.

### Sample preparation

A new stock solution of 1 mM L-tryptophan was freshly prepared for each day of measurements and was further diluted to 2–10 μM in double distilled water (Barnstead EASYpure UV/UF, reagent grade water system), TRIS (Trizma base), HEPES, phosphate buffer (NaH_2_PO_4_, Na_2_HPO_4_) or NaCl solution. The pH was set to pH 7.4 with NaOH and HCl, if not stated otherwise. Ascorbic acid and potassium iodide were added in the concentrations stated in the text. Spider silk protein (kind gift from Dr. N. Kronqvist and Prof. J. Johansson, Karolinska Institute, Stockholm, Sweden) was dissolved in 20 mM TRIS buffer to 4 μM concentration and the pH was varied from pH 7.5–5.0 with NaOH and HCl. All chemicals were purchased from Sigma Aldrich. Deoxygenation experiments were performed in a sealed container, where the solution was bubbled with nitrogen gas for twenty minutes before measurements. During the experiment, a low flow of nitrogen was applied over the sample to avoid re-oxygenation.

## Additional Information

**How to cite this article**: Hevekerl, H. *et al.* Fluorescence-based characterization of non-fluorescent transient states of tryptophan – prospects for protein conformation and interaction studies. *Sci. Rep.* **6**, 35052; doi: 10.1038/srep35052 (2016).

## Supplementary Material

Supplementary Information
