# Supplementary material for: Fluorescence-based characterization of non-fluorescent transient states of tryptophan – prospects for protein conformation and interaction studies
Source: Sci Rep. 2016 Oct 17;6:35052. doi: 10.1038/srep35052 (PMC5066179; doi:10.1038/srep35052)
Supplement: Supplementary Information [file srep35052-s1.pdf]

Supplementary Information:

Fluorescence-based characterization of non-fluorescent transient states of tryptophan – prospects for protein conformation and interaction studies

Heike Hevekerl[1], Johan Tornmalm[1], Jerker Widengren[1]

[1] Royal Institute of Technology (KTH), Dept Applied Physics, Experimental Biomolecular Physics, Albanova Univ Center, 106 91 Stockholm, Sweden

Supplementary Table 1: Fitted parameter values

I_{exc} [kW/cm^2]	k_{isc} [us^{-1}]	k_t [us^{-1}]	k_{ox1} [us^{-1}]	k_{red} [us^{-1}]	k_{ox2} [us^{-1}]
4.8	28 (fixed)	0.71 (global)	8.2	0.011 (global)	0.0020 (global)
9.2			7.8		
14			9.4		
17			9.3		
28			7.9		
65			5.7		

Power series: 40 mM TRIS, pH 7.4. Lifetime = 3 ns (fixed). Plotted in Fig. 2.

Oxygen	k_{isc} [us^{-1}]	k_t [us^{-1}]	k_{ox1} [us^{-1}]	k_{red} [us^{-1}]	k_{ox2} [us^{-1}]
21 % (Air)	28 (global)	0.74	7.4 (global)	0.0093 (global)	0.0017 (global)
0 % (Argon)		0.094			

Deoxygenation: 28 kW/cm^2, 40 mM TRIS, pH 7.4. Lifetime = 3 ns (fixed). Plotted in Fig. 2 (inset).

AA [mM]	k_{isc} [us^{-1}]	k_t [us^{-1}]	k_{ox1} [us^{-1}]	k_{red} [us^{-1}]	k_{ox2} [us^{-1}]
0	28 (fixed)	0.74 (fixed)	10 (global)	0.016	0.0039 (global)
0.3				0.025	
0.8				0.050	
3				0.11	
5				0.14	
20				0.42	

Ascorbic acid titration: 17 kW/cm^2, 40 mM TRIS, pH 7.4. Lifetime = 3 ns (fixed). Plotted in Fig. 3a.

KI [mM]	K10 [us^{-1}]	k_{isc} [us^{-1}]	k_t [us^{-1}]	k_{ox1} [us^{-1}]	k_{red} [us^{-1}]	k_{ox2} [us^{-1}]
0	328 + 3.9 [KI] - (kisc - 28) [a]	28	0.74 + 0.0029 [KI]	5.24 (global)	0.013	0.0023 (global)
0.5		31			0.017	
5		42			0.022	
20		62			0.022	
100		140			0.031	

Potassium iodide titration: 65 kW/cm^2, 40 mM TRIS, pH 7.4. Plotted in Fig. 3b. ([a] k_{isc} of 28 us^{-1} without KI, as above)

pH	Lifetime [ns]	k_{isc} [us^{-1}]	k_t [us^{-1}]	k_{ox1} [us^{-1}]	k_{red} [us^{-1}]	k_{ox2} [us^{-1}]
1.9	2.3	15 (global)	0.74 (fixed)	8.6 (global)	0.013 (global)	0.0021 (global)
2.7	2.5					
5.6	2.4					
7.7	2.8					
8.8	4.6					
10	8.5					
11	9.0					
12	7.6					

pH titration: 14 kW/ cm^2, 40 mM TRIS. Plotted in Fig. 4a.

HEPES [mM]	k_{isc} [us^{-1}]	k_t [us^{-1}]	k_{ox1} [us^{-1}]	k_{red} [us^{-1}]	k_{ox2} [us^{-1}]	k_0^+/k_0^-	k_1^+/k_1^-	k_{prot0} [us^{-1}]	k_{prot1} [us^{-1}]
0	28 (fixed)	0.74 (fixed)	8.2 (global)	0.0092	0.0024 (global)	8.7 (global)	0.15 (global)	0.51	49
0.4				0.010				0.85	69
2				0.014				2.5	200
4				0.015				4.2	270
40				0.020				32	410

Buffer concentration: 89 kW/cm^2, pH 1.9. Lifetime = 3 ns (fixed). Plotted in Fig. 4b.

pH	k_{isc} [us^{-1}]	k_t [us^{-1}]	k_{ox1} [us^{-1}]	k_{red} [us^{-1}]	k_{ox2} [us^{-1}]
5.0	99 (global)	0.34	16 (global)	0.0073 (global)	0.0011
6.0		0.30			0.0013
6.2		0.30			0.0016
6.4		0.26			0.0015
6.6		0.18			0.0022
6.8		0.15			0.0026
7.2		0.14			0.0029
7.5		0.13			0.0025

Spider silk, pH titration: 65 kW/cm^2, 40 mM TRIS, pH 7.4. Plotted in Fig. 5.
